# CRISPR/Cas9-mediated gene deletion of the *ompA* gene in symbiotic *Cedecea neteri* impairs biofilm formation and reduces gut colonization of *Aedes aegypti* mosquitoes

**DOI:** 10.1371/journal.pntd.0007883

**Published:** 2019-12-02

**Authors:** Shivanand Hegde, Pornjarim Nilyanimit, Elena Kozlova, Enyia R. Anderson, Hema P. Narra, Sanjeev K. Sahni, Eva Heinz, Grant L. Hughes

**Affiliations:** 1 Departments of Vector Biology and Tropical Disease Biology, Liverpool School of Tropical Medicine, Liverpool, United Kingdom; 2 Center of Excellence in Clinical Virology, Chulalongkorn University, Bangkok, Thailand; 3 Department of Pathology, University of Texas Medical Branch, Galveston, Texas, United States of America; 4 Department of Vector Biology and Department of Clinical Sciences, Liverpool School of Tropical Medicine, Liverpool, United Kingdom; Universita degli Studi di Pavia, ITALY

## Abstract

**Background:**

Symbiotic bacteria are pervasive in mosquitoes and their presence can influence many host phenotypes that affect vectoral capacity. While it is evident that environmental and host genetic factors contribute in shaping the microbiome of mosquitoes, we have a poor understanding regarding how bacterial genetics affects colonization of the mosquito gut. The CRISPR/Cas9 gene editing system is a powerful tool to alter bacterial genomes facilitating investigations into host-microbe interactions but has yet to be applied to insect symbionts.

**Methodology/Principal findings:**

To investigate the role of bacterial genetic factors in mosquito biology and in colonization of mosquitoes we used CRISPR/Cas9 gene editing system to mutate the outer membrane protein A (*ompA*) gene of a *Cedecea neteri* symbiont isolated from *Aedes* mosquitoes. The *ompA* mutant had an impaired ability to form biofilms and poorly infected *Ae*. *aegypti* when reared in a mono-association under gnotobiotic conditions. In adult mosquitoes, the mutant had a significantly reduced infection prevalence compared to the wild type or complement strains, while no differences in prevalence were seen in larvae, suggesting genetic factors are particularly important for adult gut colonization. We also used the CRISPR/Cas9 system to integrate genes (antibiotic resistance and fluorescent markers) into the symbionts genome and demonstrated that these genes were functional *in vitro* and *in vivo*.

**Conclusions/Significance:**

Our results shed insights into the role of *ompA* gene in host-microbe interactions in *Ae*. *aegypti* and confirm that CRISPR/Cas9 gene editing can be employed for genetic manipulation of non-model gut microbes. The ability to use this technology for site-specific integration of genes into the symbiont will facilitate the development of paratransgenic control strategies to interfere with arboviral pathogens such Chikungunya, dengue, Zika and Yellow fever viruses transmitted by *Aedes* mosquitoes.

## Author summary

Microbiota profoundly affect their host but few studies have investigated the role of bacterial genetics in host-microbe interactions in mosquitoes. Here we applied the CRISPR/Cas9 gene editing system to knockout a membrane protein in *Cedecea neteri*, which is a dominant member of the mosquito microbiome. The mutant strain had an impaired capacity to form biofilms, infected larvae and adults at lower titers, and had a reduced prevalence in adults. The lower prevalence in adults, but not larvae, likely reflects the difference in the modes of bacterial acquisition from the larval water of these two life stages. Importantly from an applied perspective, we also demonstrated that this editing technology can be harnessed for site-specific integration of genes into the bacterial chromosome. In proof-of-principle studies we integrated either a fluorescent protein or gene conferring antibiotic resistance into the bacterial genome and showed these transgenes were functional in mosquitoes. The specificity, flexibility, and simplicity of this editing approach in non-model bacteria will be useful for developing novel symbiotic control strategies to mitigate the burden of arthropod-borne disease.

## Introduction

Mosquitoes harbor a community of microbes within their guts. In general, the gut-associated microbiome of mosquitoes tends to have low species richness but can differ greatly between individuals and habitats [1–8]. Importantly, these microbes can modulate many host phenotypes, several of which can influence vectorial capacity [9–11]. As such, it is imperative that we understand how the microbiome is acquired and maintained within mosquito vectors. While environmental factors unquestionably influence the mosquito microbiome composition and abundance [2–4, 8], studies are elucidating the role of microbial interactions [5, 7, 12, 13] and host genetic factors [14–18] in shaping the microbiome. However, we have a poor understanding of bacterial factors that influence colonization of the mosquito gut and this is likely an underappreciated force influencing host-microbe interactions in mosquitoes.

In other invertebrates, several bacterial genes have been implicated in gut colonization. For example, a genome wide screen exploiting transposon-sequencing found a suite of genes from the bacterium *Snodgrasselia alvi* involved in colonization of the honey bee gut [19]. These bacterial genes were classified into the broad categories of extracellular interactions, metabolism, and stress response [19]. Knockout of a purine biosynthesis gene in *Burkholderia* impaired biofilm formation and reduced bacterial colonization rates in a bean bug [20]. Biofilm formation was also shown to play a role in virulence of pathogenic *Pseudomonas* in artificial infections of *Drosophila*, with strains that lacked the capacity to form biofilms being more virulence to the host, although a hyperbiofilm strain was less virulent than the wild type (WT) strain [21]. In other blood feeding invertebrates, bacterial genetics also appears critical for host colonization. Knockout of the type II secretion system in *Aeromonas veronii* reduced infection in *Hirudo verbena* leeches [22]. In tsetse flies, the outer-membrane protein A (*ompA*) gene of *Sodalis glossinidius* is essential for symbiotic interactions [23]. *Sodalis* mutants lacking the *ompA* gene poorly colonized the fly gut compared to the WT symbionts [23], likely due to the mutant strains reduced capacity to form biofilms [24]. Heterologous expression of the *ompA* gene from pathogenic *Escherichia coli* in *Sodalis* mutants induced mortality in the fly implicating this gene as a virulence factor in pathogenic bacteria [23]. Taken together, these studies suggest that bacterial genetic factors are critical for host colonization of invertebrates and that biofilm formation facilitates symbiotic associations in insects.

In mosquitoes, few studies have investigated how bacterial genetics affect gut colonization. However, evidence from experimental evolution studies suggests bacterial genetics plays a critical role. In two separate studies, *Enterobacter* was selected for increased persistence in the gut of *Anopheles gambiae* mosquitoes, the major malaria vector in sub-Saharan Africa, by repeatedly infecting mosquitoes with strains that persisted in the gut for longer periods of time [25, 26]. Transcriptomics comparisons of effective and ineffective colonizers in liquid media identified 41 genes that were differentially expressed between these two strains [26], further implicating the importance of bacterial genetics in mosquito infection, however the role of these genes in colonization of the mosquito gut has not been resolved. In a separate study, *in vitro* screening of a transposon mutant library of *Enterobacter* identified a *waaL* gene mutant that was insensitive to oxidative stress [27]. The *waaL* gene encodes an O antigen ligase which is needed for attachment of the O antigen to lipopolysaccharide. The mutant was found to have lower rates of colonization of the midguts of *Anopheles* mosquitoes [27].

Gene knockouts approaches in bacteria provide compelling evidence of the role of bacterial genes in host-microbe interactions [22–24, 27–29]. In general, most studies use transposon mutagenesis for gene knockout, which requires screening of the mutant library. A targeted gene knockout approach is highly desirable to investigate the functionality of bacterial genes in host-microbe interactions. In the past few years, the CRISPR/Cas9 gene editing system has been employed to modify bacterial genomes [30–32]. While much of the work has been done in model bacterial species [31–37], editing approaches have expanded into non-model bacterial systems [38–43]. Despite this expansion, the approach has been used less frequently for host-associated microbes [39, 44], and rarely for arthropod symbionts. In the vector biology field, gene knockout approaches can be used to interrogate the role of bacterial genes responsible for host-microbe interactions, whilst the ability to integrate genes into the bacterial symbiont genome has great potential for applied paratransgenic control strategies [10, 45–47]. To date, manipulation of non-model symbionts that associate with insect vectors has been accomplished by plasmid transformation [48–55] or stable transformation of the genome using transposons or integrative plasmids [56–63], but the use of CRISPR/Cas9 gene editing in insect gut symbionts has yet to be accomplished. For paratransgenic strategies, stable site-specific integration of transgenes into the symbiont genome is critical. Therefore, the application of CRISPR/Cas9 gene editing technology to non-model bacteria that associate with insect vectors will stimulate research in this field.

We therefore undertook studies to develop CRISPR/Cas9 genome editing approaches in *Cedecea neteri* isolated from *Aedes* mosquitoes. We used the Scarless Cas9 Assisted Recombineering (no-SCAR) method to disrupt the *ompA* gene of the non-model *C*. *neteri* [35]. After characterization of the mutant *in vitro*, we examined the role of the *ompA* gene in host-microbe interactions by re-infecting bacteria into mosquitoes in a mono-association. To demonstrate that the CRISPR/Cas9 gene-editing system could be useful for applied symbiotic control approaches we inserted genes conferring antibiotic resistance or a fluorescent protein into the bacterial genome and re-infected the altered strains back into mosquitoes. Our result sheds insights into the role of the *ompA* gene in host-microbe interactions in *Ae*. *aegypti* and confirm that CRISPR/Cas9 gene editing can be a powerful tool for genetic manipulation of native gut-associated microbes of mosquitoes.

## Results

### *C*. *neteri* biofilm formation in *Ae*. *aegypti* guts

**Fig 1:**
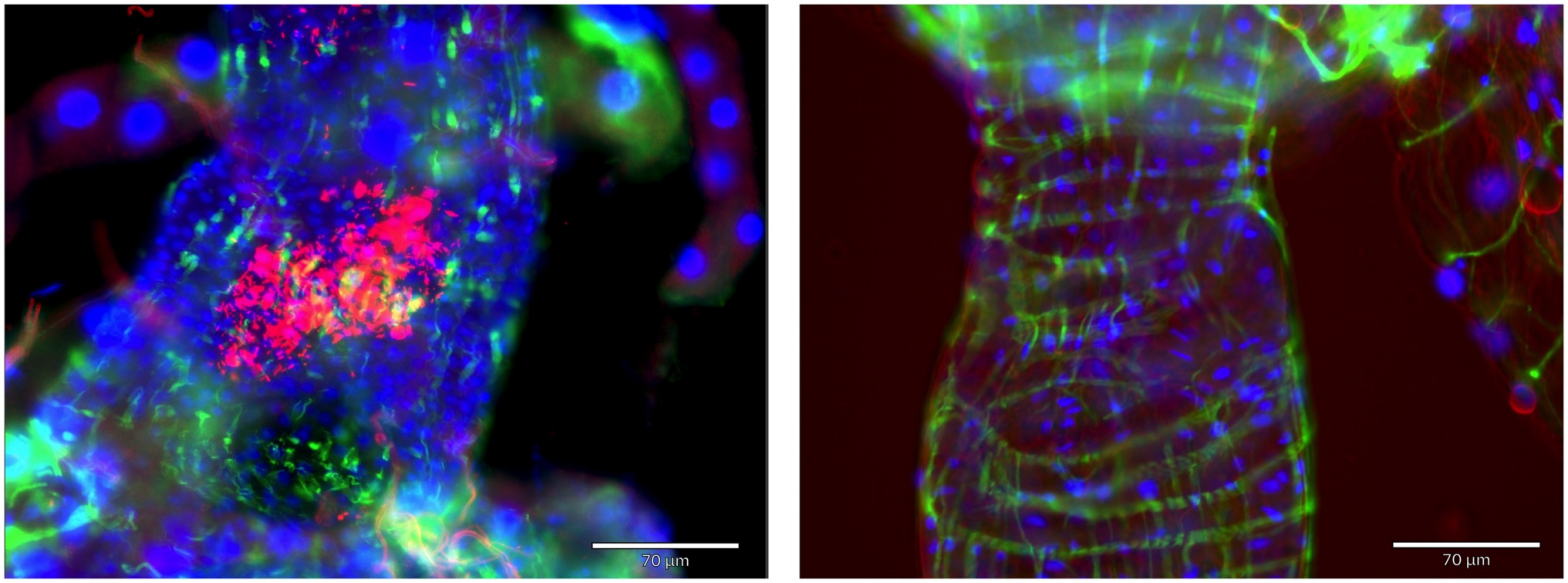
Midgut infection of *C*. *neteri* and *E*. *coli* in mono-associations of *Aedes* mosquitoes. *C*. *neteri* forms a biofilm in the gut of 3–4 day old *Ae*. *aegypti* adult mosquitoes (left) while no bacteria were observed in the gut of mosquitoes reared with *E*. *coli* under gnotobiotic conditions (right). Bacteria possessed the pRAM-mCherry plasmid, which expresses the mCherry fluorescent protein and conferred resistance to kanamycin. Blue–host nuclei stained by DAPI. Green–host actin cytoskeleton stained with phalloidin. The scale bar is 70 μm.

Over the course of conducting mono-axenic infections in *Ae*. *aegypti* mosquitoes with a *Cedecea* symbiont, we repeatedly observed a conglomeration of bacterial cells in the anterior and posterior midgut (Fig 1, S1 Fig) that had a similar appearance to biofilms observed in the guts of other insects [21, 24]. We also infected mosquitoes with the *E*. *coli* BL21(DE3) lab strain as a control, but we did not see any evidence of infection (Fig 1, S1D–S1F Fig) although infection with this bacterium enabled mosquito development [64]. The *E*. *coli* BL21(DE3) lab strain does not have the capacity to form biofilms [65], possibly explaining its inability to infect mosquitoes. We therefore set out to examine the role of bacterial genetics in biofilm formation and host colonization of gut-associated bacteria of *Aedes* mosquitoes. We used multilocus sequence typing (MLST) to confirm the species of our isolate, which indicated the bacterium was *C*. *neteri* (S2 Fig). Several genes have been implicated in biofilm formation [21, 24], but we chose to knockout the *ompA* gene of *C*. *neteri* given that this gene has been demonstrated to influence biofilm formation and gut colonization of *Sodalis* [23, 24], an *Enterobacteriaceae* symbiont of tsetse flies. We used the CRISRP/Cas9 genome editing system to mutate the symbiont genome to demonstrate this approach could be employed for non-model symbiotic bacteria that associate with mosquitoes.

### Genome editing in *C*. *neteri* bacteria isolated from mosquitoes

**Fig 2:**
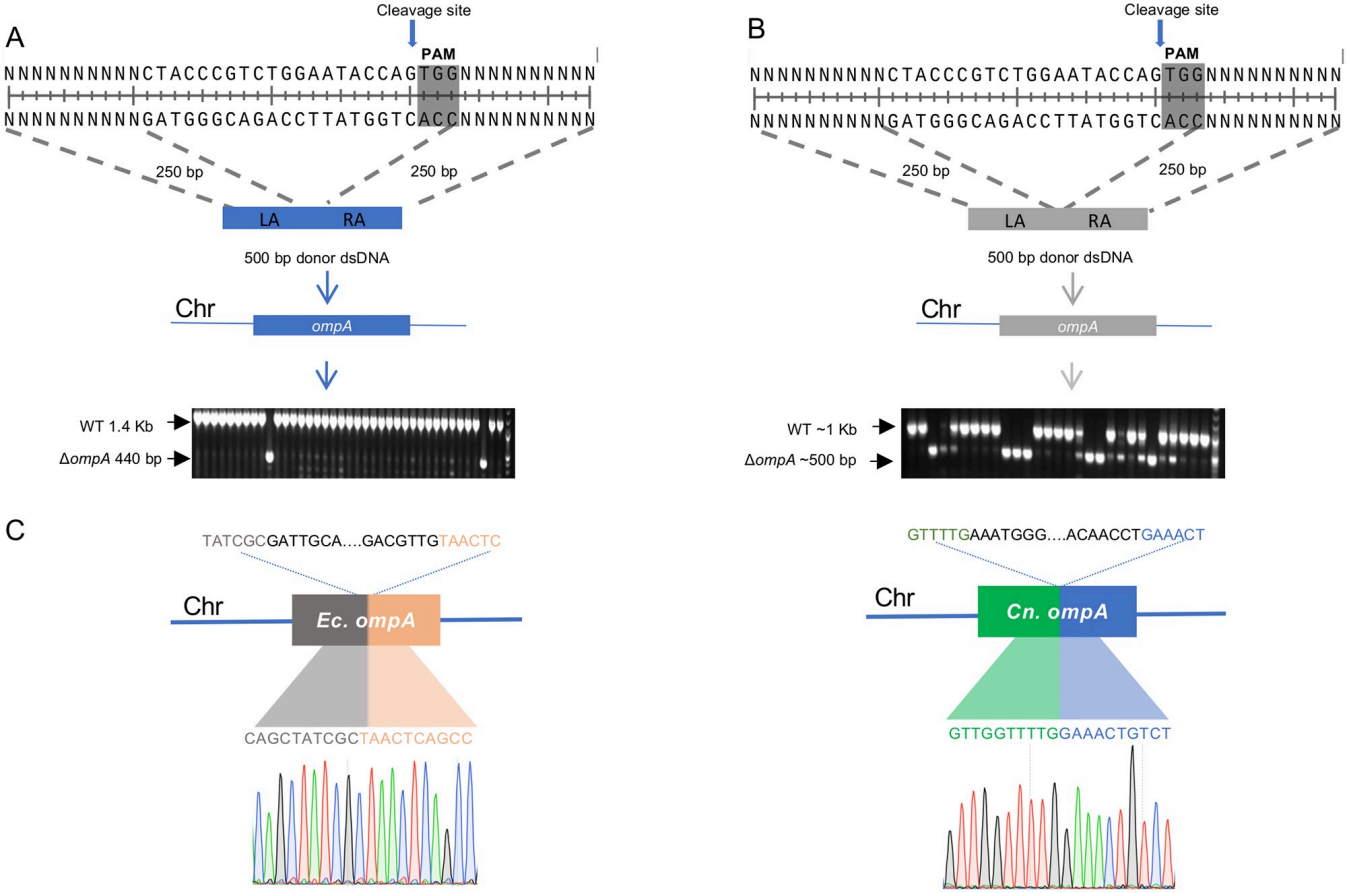
CRISPR/Cas9 genome editing in bacteria. A schematic of the editing approach and screening of putative mutants in (A) *E*. *coli* and (B) *C*. *neteri*. A ~1kb fragment of *E*. *coli* BL21(DE3) was deleted using no-SCAR protocol. The 250 bp of the left arm (LA) and right arm (RA) was assembled to generate the 500 bp donor DNA. The transformants were screened via colony PCR with primers binding in regions flanking the deletion. Similar to the strategy employed in *E*. *coli*, the knockout of the *ompA* gene from *C*. *neteri* isolated from the mosquito gut was created by deleting the 598 bp fragment. The grey area indicates the PAM site in the *ompA* gene and arrow shows the cleavage site in the genome. (C) The sequence of the *ompA* mutation in *E*. *coli* and *C*. *neteri* was confirmed by Sanger sequencing. The sequence above the gene within the dotted line has been deleted. The chromatogram shows the 10 bp flanking the deletion.

To edit the *Cedecea* isolate that resides within the gut of *Aedes* mosquitoes, we employed the no-SCAR gene editing approach that had been developed in *E*. *coli* [35]. To optimize the approach in our hands, we performed initial experiments in *E*. *coli* to delete a ~1 kb region of the *ompA* gene (Fig 2A). As the no-SCAR approach exploits the λ-Red recombineering system to repair double stranded breaks, we transformed bacteria with a double stranded DNA template that had regions of homology flanking the gRNA site (250 bp for each arm). Using this approach, we successfully deleted a 1001 bp fragment of the *ompA* gene. From the colonies we screened, we saw an editing at a frequency of 6.25% (N = 48) (Fig 2A). For *C*. *neteri*, we altered our editing procedure to delete a 598 bp fragment from the *ompA* gene. This was done to enhance the efficiency of obtaining mutants [35] and accommodate the PAM site which was at a different location in the *ompA* gene in *C*. *neteri*. Using a donor template designed for the *C*. *neteri ompA* gene that had flanking homology arms of similar length as the previous experiment done in *E*. *coli*, we obtained mutant knockouts at a rate of 32% (N = 50) (Fig 2B). For both bacterial species, Sanger sequencing across the integration site indicated the deletion occurred at the expected loci in the bacterial genome (Fig 2C; S1 Appendix).

### Characterization of the *C*. *neteri ompA* mutant

**Fig 3:**
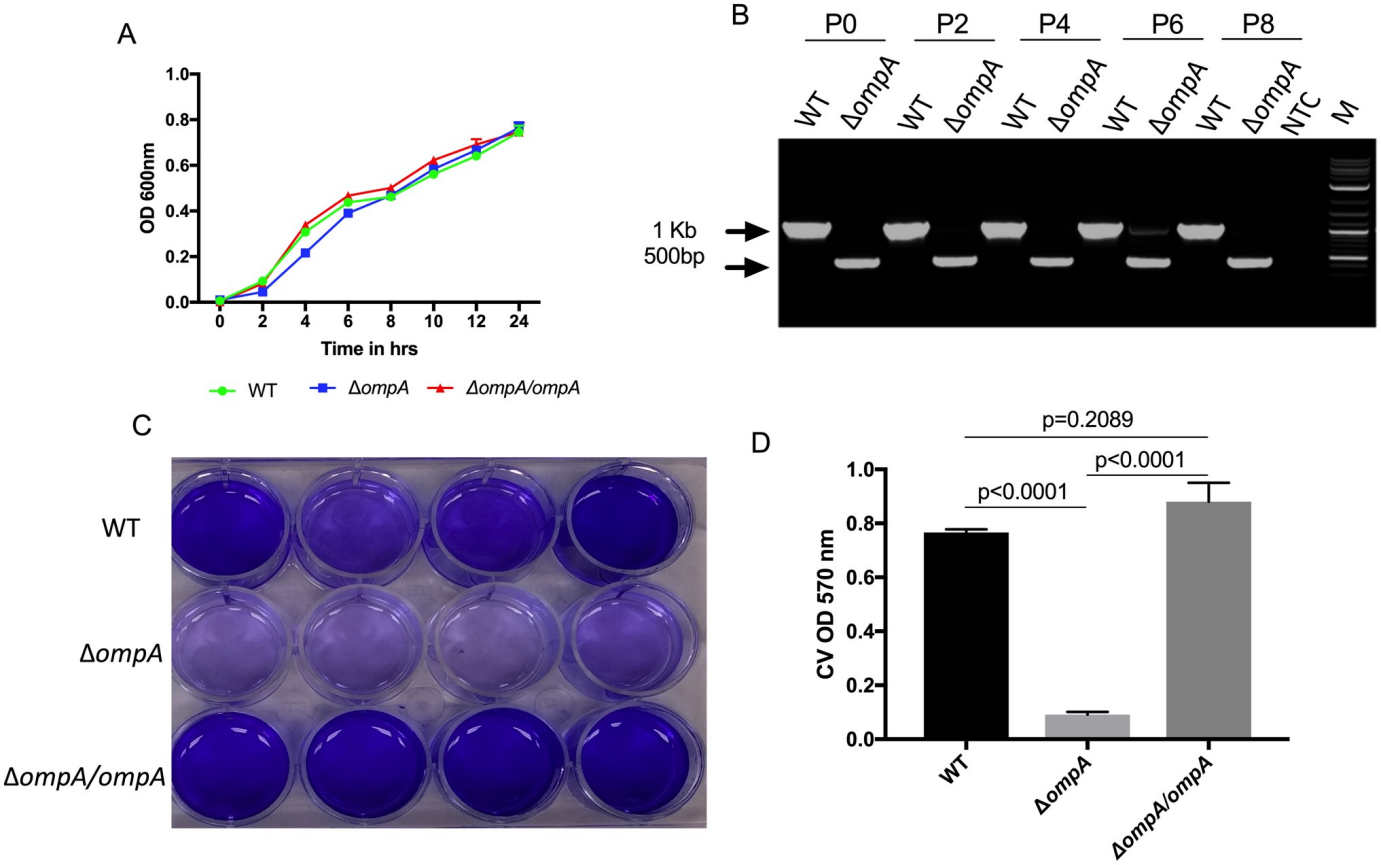
*In vitro* characterization of the *ompA* mutation. (A) The *C*. *neteri ΔompA* mutant had a similar growth rate compared to both the WT and the Δ*ompA/ompA* complement in liquid LB media. Five technical replicates were used to create growth curves. (B) The stability of mutant was evaluated *in vitro* by continuous subculturing in LB media. Genomic DNA from alternative subcultures was used as template for PCR using primers that amplified across the deletion. The stability assay was repeated twice. Two separate gel images were merged to create figure 3B (passage 8 was run on a separate gel to passages 0–6). (C) Biofilm formation was assessed using the CV biofilm assay for the WT, Δ*ompA* mutant and the Δ*ompA/ompA* complement. Two biological replicates were completed. (D) Quantification of the relative biofilm formation normalized by the number of bacteria per well (N = 3). Error bars represent standard error. The assay was repeated twice.

We quantified the growth rates of the Δ*ompA* mutant in comparison to the WT *C*. *neteri* and the Δ*ompA/ompA* complement in liquid LB media. We saw minimal differences in the growth between the WT, the Δ*ompA* mutant or the Δ*ompA/ompA* complement (Fig 3A). To examine the stability of the deletion, we subcultured the Δ*ompA* mutant on LB media for 10 generations and performed PCR to amplify across the deletion. At alternative generations, PCR analysis indicated the deletion was present indicating genomic stability at this site (Fig 3B).

Previously, *ompA* has been shown to be important in biofilm formation as *Sodalis* deletion mutants were unable to form biofilms [24]. Therefore, we characterized *in vitro* biofilm formation using the crystal violet (CV) biofilm assay. From visual inspection, it was clear the Δ*ompA* mutant had distinctly less biofilm deposition compared to either the WT or the Δ*ompA/ompA* complement (Fig 3C). After quantification and normalization to account for any difference in growth between the strains, biofilm formation was confirmed to be significantly different between the Δ*ompA* mutant and the WT or complement (Fig 3D; Tukey’s multiple comparisons test, P < 0.0001). There was no significant difference between the WT and the Δ*ompA/ompA* complement (Tukey’s multiple comparisons test P = 0.2).

### The role of *ompA* gene in mosquito infection

**Fig 4:**
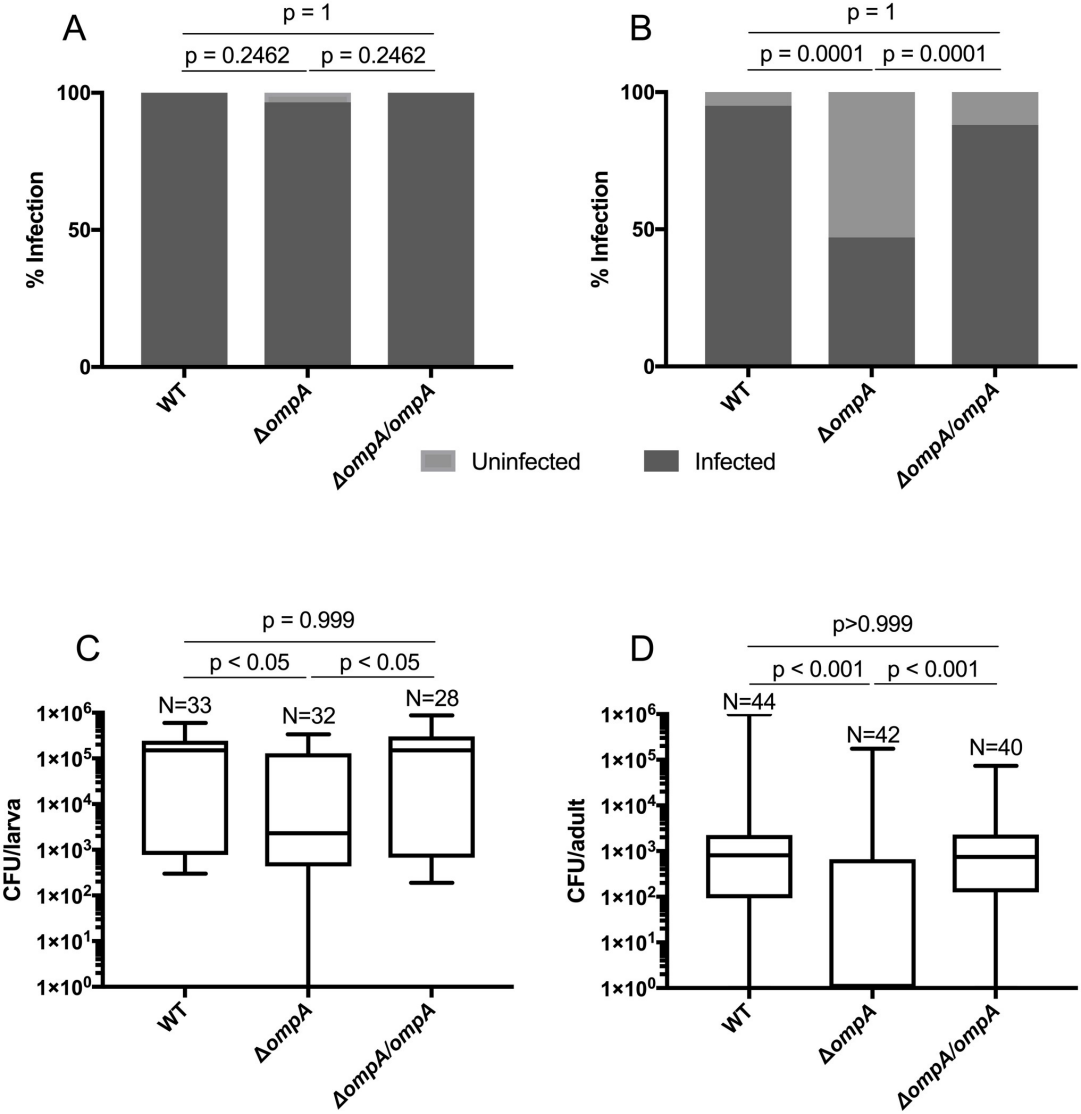
The Δ*ompA* mutant poorly infected mosquitoes. Infection of *C*. *neteri* strains (WT, Δ*ompA* mutant and Δ*ompA/ompA* complement; the former two possessed the pRAM-Cherry plasmid while the latter possessed the pRAM-Cherry-Ent-*OmpA* plasmid) reared in a mono-association using a gnotobiotic rearing approach for larvae (A and C) and adults (B and D). L4 larvae and 3–4 days post emergence adults were screened for bacterial load by plating on selective LB media with kanamycin to quantify the bacteria. The prevalence of infection (number of mosquitoes infected) between the treatments was calculated comparing the number of infected to uninfected larvae (A) or adults (B). Density of bacteria (CFU/mosquito) in larvae (C) and adults (D). The assay was repeated twice. Results display pooled data from each independent replicate. Box and whiskers show the median, the 25^th^ and 75^th^ percentiles and the minimum and maximum values.

**Fig 5:**
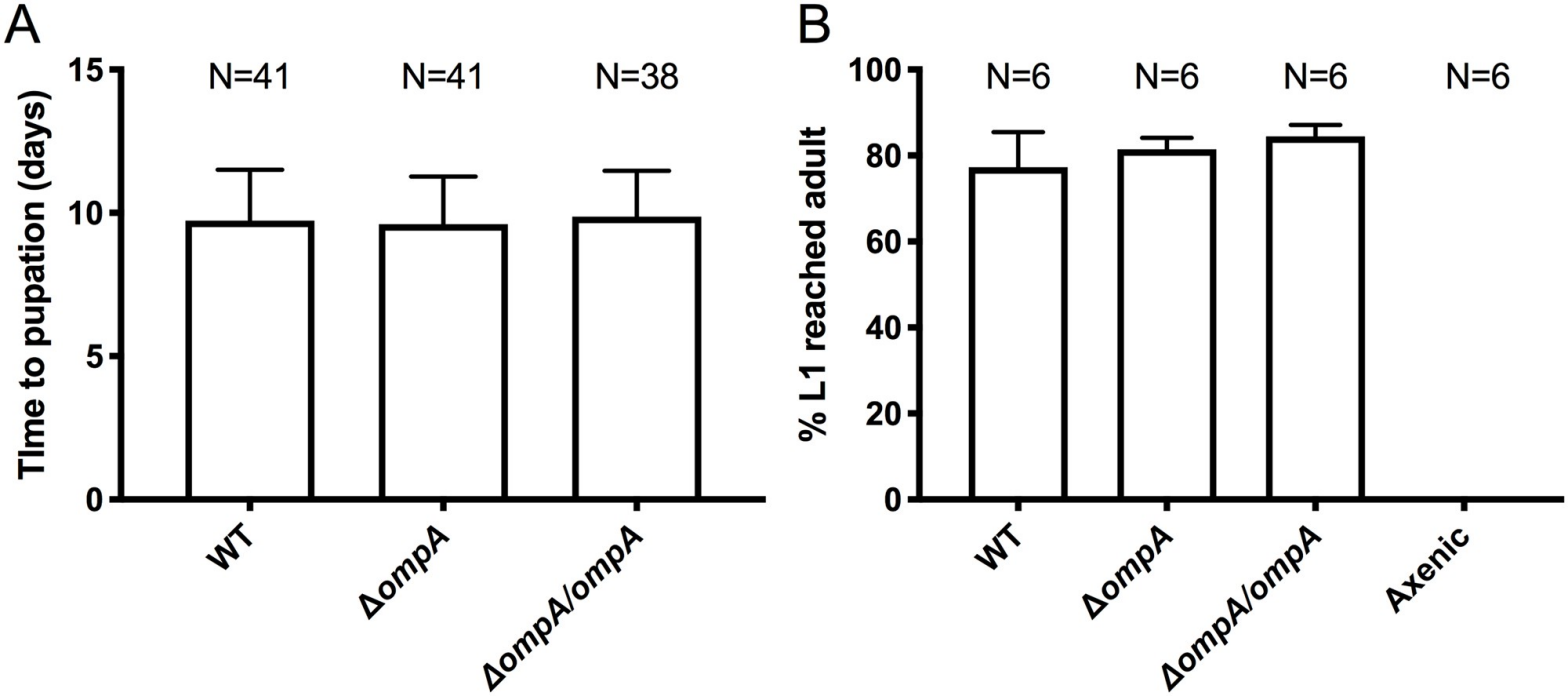
The Δ*ompA* mutant does not affect growth rates or development of mosquitoes. The growth rate (time to pupation) (A) and development (percentage of L1 larvae to reach adulthood) (B) was observed in mosquitoes infected with *C*. *neteri* strains (WT, Δ*ompA* mutant and Δ*ompA/ompA* complement) reared in a mono-association. The experiment was done twice with a minimum of 15 individuals. Sample size for panel A indicates number of individuals, while for B indicates the number of replicate flasks. Each flask has 20 mosquitoes.

To examine the importance of the *ompA* gene on bacterial colonization of mosquitoes, we infected *Ae*. *aegypti* mosquitoes in a mono-association under gnotobiotic conditions [64]. This infection method was used to avoid other gut-associated microbes influencing host colonization rates [7] and it also enabled straightforward quantification of introduced bacteria by measuring colony forming units (CFUs). No significant changes were seen in the prevalence of infection (number of mosquitoes infected) in the larval stage (Fig 4A, Fisher’s exact test; WT compared to Δ*ompA* P = 0.24 and Δ*ompA* compared to Δ*ompA/ompA* P = 0.24) with rates of infection consistently high (WT 100%, Δ*ompA* 96%, and Δ*ompA/ompA* 100%). In adults, the prevalence of infection was significantly different (Fig 4B, Fisher’s exact test; WT compared to Δ*ompA* P < 0.0001 and Δ*ompA* compared to Δ*ompA/ompA* P < 0.0001), with only 45% of adults infected by the Δ*ompA* mutant compared to 95% and 88% by the WT and Δ*ompA/ompA* complement, respectively. In larvae, we saw a significant reduction in bacterial titer in the mutant compared to both the WT (Kruskal-Wallis test with Dunn’s test; P < 0.05) and the Δ*ompA/ompA* complement (Kruskal-Wallis test with Dunn’s test; P < 0.05) (Fig 4C) with median values of 1.5x10^5^, 2.3x10^4^, and 1.5x10^5^ for the WT, Δ*ompA*, and Δ*ompA/ompA* complement respectively. Similarly, in adults, there was a significant reduction in bacterial infection in the Δ*ompA* mutant compared to either the WT or Δ*ompA/ompA* complement (Kruskal-Wallis test with Dunn’s test; P < 0.001) (Fig 4D), with median value of 8.1x10^2^, 0, and 7.5x10^2^ for the WT, Δ*ompA*, and Δ*ompA/ompA* complement respectively. However, when considering only the infected mosquitoes for analysis, we saw no significant difference between the treatments (S3 Fig, Kruskal-Wallis test with Dunn’s test; P > 0.99). For both the larvae and adult density quantifications, the non-parametric test (Kruskal-Wallis test) was used due to non-normal distribution of data (Sharpiro-Wilks test; P<0.001). We also monitored the growth rates of mosquitoes administered with the WT, Δ*ompA* mutant and Δ*ompA/ompA* complement. No significant differences were seen in the time to pupation (Fig 5A) or percentage of first instar larvae that reached adulthood (Fig 5B) between any of the bacterial strains.

### Integration of genes into the *C*. *neteri* chromosome

**Fig 6:**
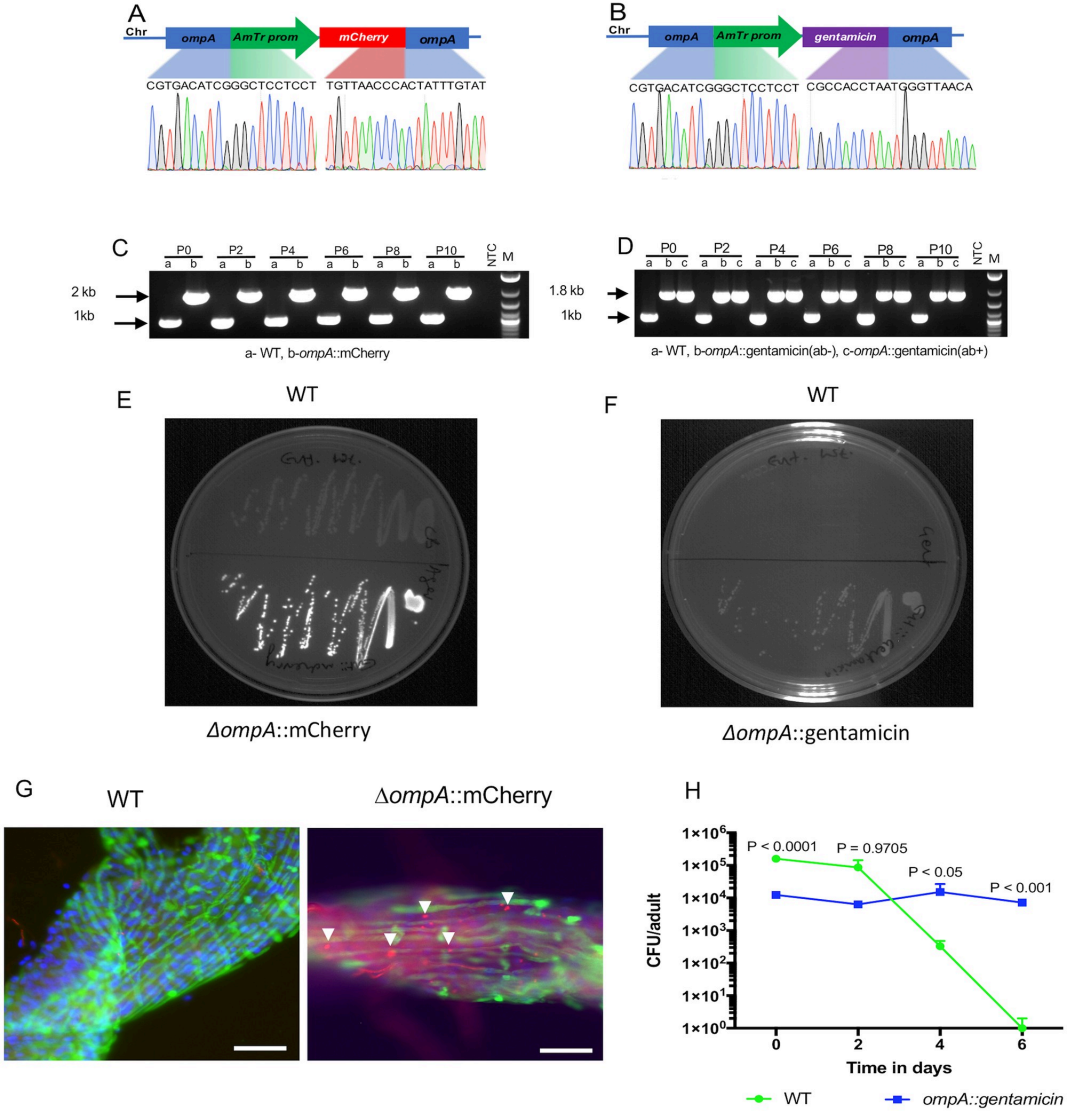
Integration of mCherry and gentamicin into the *C*. *neteri* genome. Sanger sequence across the integration site, stability of the inserted gene and *in vitro* expression of the inserted gene for the Δ*ompA*::mCherry (A-C) and the Δ*ompA*:: gentamicin (B-D) strains. The chromatogram shows the sequence spanning the inserted sites. Strains were continually subcultured for 10 passages and PCR was done to examine the stability of the insert (C; Δ*ompA*::mCherry plus WT, D; Δ*ompA*::gentamicin passaged with (ab+) or without (ab-) gentamicin plus WT). mCherry fluorescence (E) or ability to grow on selective media containing gentamicin (F) confirmed the expression of the transgene *in vitro*. Mosquitoes were inoculated with the *C*. *neteri* strains to confirm expression of the transgene *in vivo*. Dissected midgut infected with Δ*ompA*::mCherry (left) or negative control (right; WT bacteria without expression plasmid) (G). Midguts were stained with phalloidin (green) and DAPI (blue). The scale bar is 30 μM. The WT and Δ*ompA*::gentamicin *C*. *neteri* strains were fed to adult mosquitoes for 3 days in a sugar meal before gentamicin was administered to mosquitoes in sugar without bacteria (H). Mosquitoes were collected every second day and CFUs assessed. Pairwise comparisons were conducted at each time point using a T test.

We undertook experiments to demonstrate the CRISPR/Cas9 gene-editing approaches can be used to integrate genes into the chromosome of non-model bacteria that associate with mosquitoes. We created two independent transgenic strains that had either a gene encoding mCherry fluorescence or a gene encoding resistance to the antibiotic gentamicin inserted into the bacterial chromosome. Before undertaking these integration experiments we confirmed that *C*. *neteri* was susceptible to gentamicin. These genes were integrated into the genome using the same gRNA that was used for deletional mutagenesis (S1 Table), and as such, these insertions also disrupted the *ompA* gene. Sequencing across the integration site indicated the insertion of these genes occurred within the *ompA* gene and thereby disrupted its function (Fig 6A and 6B). Continual subculturing was undertaken for both strains and molecular analysis indicated the stability of these lines for ten generations (Fig 6C and 6D). Expression of mCherry fluorescence and growth of the Δ*ompA*::gentamicin strain on media containing gentamicin demonstrated the integrated genes were functional *in vitro* (Fig 6E and 6F).

To examine the functionally of the integrated genes in the mosquito we administered either WT, Δ*ompA*::mCherry, or Δ*ompA*::gentamicin to conventionally reared 3–4 day old adult female *Ae*. *aegypti* in a sugar meal for 3 days or larvae cleared of their microbiota. For gnotobiotic infection we used bacteria expressing mCherry from a plasmid. The dissected gut from 3–4 day old adults showed a higher percentage of WT bacteria compared to either of the integrated mutants. After screening midgut samples from each treatment, we found that mosquitoes infected with WT bacteria had the highest infection prevalence (69%) and that the mCherry and gentamicin knockin mutants were found only in 4% and 33% of the samples, respectively (S4 Fig, S4 Table). In addition, biofilms were seen mainly in mosquitoes infected with WT bacteria (31%) whilst midguts infected with mutants had few or no biofilms (0–2%) (S4 Fig, S4 Table). In sugar fed adult mosquitoes, Δ*ompA*::mCherry bacteria were observed in the gut of mosquitoes with a distinct punctate distribution, whereas no signal was seen in autofluorescence controls (WT *C*. *neteri* infected mosquitoes) (Fig 6G). The *C*. *neteri ompA*::gentamicin was successfully rescued from mosquitoes reared on gentamicin and stably infected mosquitoes over time at a density of approximately 1x10^4^ CFUs/mosquito. Consistent with our previous result (Fig 4B), WT bacteria initially infected mosquitoes at higher titers compared to the mutant (T test; day 0 P < 0.001). However, after 4 days rearing on antibiotic the total bacterial load in mosquitoes administered WT *C*. *neteri* was significantly reduced compared to the Δ*ompA*::gentamicin (T test; day 4 P < 0.05) while the prevalence of mosquitoes with culturable microbiota was reduced to 80%. After 6 days rearing on antibiotic, the Δ*ompA*::gentamicin density was significantly elevated compare to the WT (T test; day 6 P < 0.001) only one mosquito was infected, which had a low density infection (10 CFUs/mosquito) (Fig 6H).

## Discussion

We harnessed the CRISPR/Cas9 gene editing system to create knockout mutants in a *C*. *neteri* gut symbiont of *Aedes* mosquitoes to examine the role of bacterial genetics in biofilm formation and gut colonization. A deletion of the *ompA* gene of *C*. *neteri* decreased bacterial colonization of mosquitoes after infection in a mono-association. Strikingly, we found this effect was most pronounced in adult mosquitoes with more than half of the mosquitoes not possessing any culturable mutants, whereas there was no difference in prevalence of infection between the mutant and WT bacteria in larvae. The reduced prevalence of mutant bacteria in adults likely reflects differences in microbial colonization of each mosquito life stage. Larvae are continually subjected to bacteria in the larval water habitat while adults only have a short time frame to acquire bacteria from the aquatic environment immediately after eclosion. Alternatively, the reduced prevalence in adults could be due an impaired ability of mutant bacteria to be transstadially transmitted. Several bacterial species have been shown to exploit this process to transfer between life stages [66–69]. When only analysing adult mosquitoes where bacteria did colonize the host, we saw no differences in the density of the mutant strain compared to the WT or complement, suggesting that *ompA* is acting at the colonization stage but has minimal effect on post-colonization processes. However, when examining midguts using fluorescent microscopy, in general, we observed reduced loads of the mutant strains. When quantifying bacterial load by CFU we used whole mosquitoes. It may be possible that mutant bacteria were residing in other tissues in the adult but poorly re-infected the midgut. If this occurred, it would indicate involvement of *ompA* in transstadial transmission. The greater variability seen in the prevalence of adults compared to the larval is consistent with other sequence-based studies that indicate adult stages have greater variability in species composition of their microbiota, whereas the microbiome of immature stages is similar to the microbiota in larval water habitat [2–5, 8, 70].

Mutant bacteria colonized mosquitoes at higher densities when administered to adults as opposed to larvae. There are several possible explanations for this finding. The first relates to the method of inoculation with adults being administered bacteria in a sugar meal while larvae were exposed to bacteria in their aquatic environment. The different inoculation process itself may influence titer but also when sugar feeding, adults had the opportunity for repeated infections whereas emerging adults only had a narrow window for inoculation as they did not have further access to the larval water habitat after eclosion. The second explanation relates to differences in the microbiome of these mosquitoes. The mosquitoes inoculated as adult were reared conventionally, and as such, had an intact microbiome, while larvae reared in the gnotobiotic system only possessed the individual *Cedecea* strains that were administered. For the latter group there was no opportunity for the native WT bacteria (either of the same or different species) to rescue the mutant phenotype. In the *Sodalis*-*tsetse* system, mutant bacteria were capable of infecting flies that had an intact microbiome but were unable to infect *Sodalis*-free *tsetse* flies [23], suggesting WT *Sodalis* facilitated colonization of the mutant strain. In mono-axenic infections, the *C*. *neteri* mutant strain was able to infect *Ae*. *aegypti*, indicating that *ompA* is not essential for infection in the mosquito-*Cedecea* system.

Our results, in conjunction with studies in the *Sodalis*-tsetse system [23, 24], suggests that biofilm formation may be a strategy employed by bacteria to colonize the gut of insects. In pathogenic infections in mammals, biofilms enable bacteria to colonize new niches, promote infection, and are associated with virulence [71]. Although less is known regarding the importance of biofilm formation in insects, in an artificial *Pseudomonas-Drosophila* infection model, biofilm formation was associated with virulence and host survival [21]. In a natural symbiotic association between bean bugs and *Burkholderia*, disruption of a purine biosynthesis gene in the bacterium reduce biofilm formation and colonization of the insect [20]. In mosquitoes, gut biofilm formation could also have implications for vector competence. *Chromobacterium*, which was isolated from *Aedes* mosquitoes, produced molecules that inhibited dengue virus only when grown *in vitro* as a biofilm but not when grown in a planktonic state [72], however it is unknown if biofilm formation occurred *in vivo* in the mosquito. Our data provide evidence that biofilms occur within the gut of mosquitoes and facilitate host colonization.

Although we have shown that the *ompA* gene of *C*. *neteri* is important for host colonization, we see no evidence that deletion of this gene alters mosquito development or growth rates. This is in contrast to the *Riptortus*-*Burkholderia* symbiosis whereby mutation of the *purT* gene in *Burkholderia* resulted in reduced growth rates and reduction in body weight of the host compared to insects that were infected with the WT bacterium [20]. The difference in our study to the findings in the *Riptortus*-*Burkholderia* symbiosis could be related to different requirements of the bean bug compared to the mosquito host as well as the different genes mutated in the symbionts. Our findings are consistent with a previous study in *Ae*. *aegypti* whereby an *ompA* mutant of *E*. *coli* did not influence growth when reared in a mono-association [73]. Using a similar gnotobiotic system that exploits the ability to sterilize mosquito eggs and rescue development by nutritional supplementation, several recent reports describe approaches to create bacteria-free mosquitoes [73, 74]. Here, we reared mosquitoes in a mono-association where they were only subjected to *C*. *neteri*. However, more than half the adult mosquitoes inoculated with the Δ*ompA* mutant were not infected by bacteria, as evidenced by the inability to culture bacteria from these insects. Nevertheless, these mosquitoes had similar development and growth rates compared to mosquito possessing WT bacteria. The use of mutant bacteria that rescue development but have an impaired ability to colonize mosquitoes may provide a simple means to create axenic adult mosquitoes.

CRISPR/Cas9 gene editing has revolutionized genetic approaches in model and non-model bacteria [31–43]. However, there has been limited use of this technology in symbiotic microbes of arthropods. Here we demonstrate that editing approaches functional in *E*. *coli* can be easily applied with minimal adaptation to phylogenetically related symbiotic bacteria that are found within the guts of mosquitoes. The application of CRISPR/Cas9 genome editing to gut-associated bacteria of mosquitoes has significant applied potential. Paratransgenesis strategies are being evaluated in a range of medical and agricultural systems to mitigate pathogen transmission from insect vectors, however, most approaches engineer symbionts by plasmid transformation [49–55, 75] and where genome integration has been accomplished in symbionts [58–61], it has often been done with technologies that did not allow for site specific integration. Paratransgenic approaches suitable for use in the field will need to stably integrate genes into the bacterial genome in a manner that does not compromise bacterial fitness. Exploiting the flexibility and specificity of the CRISPR/Cas9 system to integrate genes in intergenic regions of the bacterial chromosome will undoubtedly be beneficial for these applied approaches.

In summary, we have demonstrated that the CRISPR/Cas9 gene editing system can be applied to symbiotic bacteria that associate with eukaryotic hosts to interrogate the role of bacterial genes in host-microbe associations. We created knockout and knockin mutants by deleting and disrupting the *ompA* gene of *C*. *neteri*. The knockout mutant displayed a reduced ability to form biofilms and colonize the gut of *Ae*. *aegypti* mosquitoes in a mono-association demonstrating bacterial genetic factors are important determinants that influence colonization of mosquito guts. *Aedes* mosquitoes are becoming powerful systems to investigate the genetics of host-microbe interactions given the scientific community has simple and efficient approaches to alter both the microbes (this study) and mosquito host genome [76, 77] at their disposal, as well as methods to create mono-associated mosquito lines [7, 64]. Finally, rapid, efficient, and site specific gene editing approaches for gut bacteria that associate with mosquitoes will facilitate the development of novel paratransgenic approaches to control arthropod-borne disease [57].

## Material and methods

### Bacterial and mosquito strains

*E*. *coli* BL21(DE3) (NEB) and *Cedecea neteri* strain Alb1, previous isolated from a lab-reared colony of *Ae*. *albopictus* (Galveston) mosquitoes [7], were used in this study. To further classify the gut-associated bacteria we completed multilocus sequence typing [78]. DNA from the single colony was used as a template in a PCR to amplify genes for MLST analysis (S3 Table). Amplicons were resolved on a 1% agarose gel, extracted and purified, and Sanger sequenced. The *atpD*, *infB*, *gyrB* and *rpoB* genes were aligned separately, using the species diversity as in [79] with several *Cedecea* sp. sequences and then concatenated using seaview [79]. The phylogenetic tree was constructed using iqtree [80] under the general time-reversible (GTR) model with 1000 fast bootstrap replicates, which are shown as percentage branch support values (S4 Fig). The sequences of our isolate are available under accessions (MN329096 (*atpD*), MN329097 (*gyrB*), MN329098 (*infB*), MN329099 (*rpoB*). For gene editing and mosquito infections, cultures were grown in liquid LB media at 37°C with the appropriate antibiotic unless stated otherwise. Mosquitoes were reared in the UTMB insectary under conventional conditions or in a mono-association (described below).

### CRISPR gene editing

Designing protospacer sequence and cloning: The *E*. *coli* BL21 *ompA* gene sequence was retrieved from NCBI (accession number LR536431). The *C*. *neteri* Alb1 *ompA* gene was PCR amplified and Sanger sequenced using primers (OmpA-F and OmpA-R, S3 Table), which were designed based on the *Enterobacter cloacae ompA* (accession number CP017990). Editing the *ompA* gene of *E*. *coli* and *C*. *neteri* was complete as described in Reisch and Prather [35]. Protospacer sequences for the *ompA* gene were designed using CHOPCHOP [81, 82]. To clone the protospacer sequences into pKDsgRNA-ack (S2 Table; Addgene plasmid #62654) we amplified the entire plasmid with primers that contained the protospacer sequence and this amplicon was self-ligated. This PCR was done using 0.5μM of each primer (S1 Table), 1x reaction buffer, 200μM dNTPs, 0.5U of Phire Host Start Taq polymerase (Thermo Scientific) and 200 ng of plasmid DNA as template. The cycling condition consisted of an initial denaturation step 98°C for 2 minutes, followed by 35 cycles of 98°C for 2 seconds, 58°C for 15 seconds, and 72°C for 2 minutes and 30 seconds, and then a final extension at 72°C for 10 minutes before holding at 16°C. The PCR products had a 15–17 bp overlapping sequence which was used to ligate the plasmid. The PCR product was digested with *Dpn*I to remove any template plasmid. PCR products were then ligated by transformation into *E*. *coli* harbouring the Red/ET plasmid following the REPLACR mutagenesis protocol [83], thereby creating plasmids pKDsgRNA-Ec-*omp*A-1, pKDsgRNA-Ec-*omp*A-2, pKDsgRNA-Ent-*omp*A-1, *and* pKDsgRNA-Ent-*omp*A-2 (S2 Table). Colonies were screened for the protospacer insertion by PCR and confirmed by Sanger sequencing.

### Knockout of *ompA*

The two protospacers were evaluated by transforming plasmids into either *E*. *coli* or *C*. *neteri* containing the pCas9-CR4 plasmid (S2 Table; Addgene plasmid 62655), which expressed Cas9 nuclease. Transformants were selected at 30°C on LB agar plate containing spectinomycin (50 μg/mL), chloramphenicol (34 μg/mL), and either with or without anhydrotetracycline (aTC; 100ng/mL). The escape rate was quantified by comparing colonies in the plates with or without aTC. The protospacer with a lack of or few escape mutants was used for further experiments. Colonies from the–aTC plate were grown overnight in LB broth with the appropriate antibiotic at 30°C. A 1:100 diluted overnight culture was (grown until 0.4 OD_600_) supplemented with 1.2% arabinose to induce the expression of λ-Red recombinase for 20 min. Cells were then transformed with 1–1.5 μg of double stranded donor DNA for homologous recombination. Donor DNA was created by PCR amplifying the flanking left arm (LA) and right arm (RA) from *E*. *coli* and *C*. *neteri* genomic DNA. Each arm had flanking regions of 250 bp homologous to the target DNA. The resulting fragment was assembled using Gibson assembly (NEB). The assembled product was amplified to generate full length dsDNA for transformation. Colonies were screened for mutations by colony PCR with primers flanking the integration site and positive clones were Sanger sequenced (S3 Table). Positive colonies were grown in LB broth and genomic DNA was isolated. For further validation, the flanking regions of deletion or insertions were amplified, and the PCR product Sanger sequenced.

### Insertion of mCherry and gentamicin gene into *C*. *neteri* genome

The plasmid pKDsgRNA-Ent-*ompA* was transformed into *C*. *neteri* and the gene editing procedure was repeated as described above. To generated the donor sequence for homologous recominbination the mCherry or gentamicin sequence (driven by the AmTr promoter) and each homology arm were amplified and ligated. The assembled product was amplified to generate a full length dsDNA fragment for transformation.

### Stability of insertion

The stability of the knockout Δ*ompA* mutant and the knockin *ompA*::gentamicin and *ompA*::mCherry strains was assessed in LB media. The *omp*A::mCherry and knockout Δ*ompA* mutant cultures were grown for 10 passages in LB broth. At each passage 40 μl of culture was transferred into 4ml fresh LB media. The *ompA*::gentamicin strain was grown with or without gentamicin (50 μg/mL). Genomic DNA was isolated from the 0, 2, 4, 6, 8 and 10^th^ subculture and PCR that amplified across the integration site was performed.

### Complementation of *ompA* mutant

Functional rescue of the *omp*A mutation was achieved by complementing the mutant with the WT gene. The WT *omp*A gene was amplified from *C*. *neteri* genomic DNA and cloned into the pRAM-mCherry vector [7] in front of the *ompA* promoter, thereby creating pRAM-mCherry-*Ent*-*OmpA* plasmid. The Sanger sequence-verified plasmid was transformed into the Δ*ompA* mutant, thereby generating the Δ*ompA/ompA* complement strain. Colonies that acquired the plasmid were selected on LB plates containing kanamycin (50 μg/mL).

### *In vitro* characterization of *C*. *neteri* strains

To assess the impact of the gene deletion on bacterial growth the WT, Δ*ompA* mutant and Δ*ompA/ompA* complement were grown in LB broth and the density of bacteria (OD_600_) was quantified by spectrophotometer. A 1:100 dilution of an overnight culture was inoculated into a 5 ml LB broth in a 50 ml tube and incubated at 37°C for 24 hrs. At 2, 4, 6, 8, 10, 12 and 24 hours growth was recorded at OD_600_. The biofilm assay was performed as described previously [84, 85]. Briefly, biofilm formation by *C*. *neteri* strains was quantified on polystyrene microtiter plates after 72 h of incubation at 37°C by CV staining. Three independent experiments were performed, and the data were represented as CV OD_570_ after normalizing by CFUs.

### Mosquito infections

Mono-association in *Ae*. *aegypti* mosquitoes were done using gnotobiotic infection procedure [7, 64], with slight modifications. Briefly, mosquito eggs were sterilized for 5 min in 70% ethanol, 3 min in 3% bleach+0.01% Coverage Plus NPD (Steris Corp.), 5 min in 70% ethanol then rinsed three times in sterile water. Eggs were vacuumed hatched for 30–45 min and left overnight at room temperature to hatch any remaining eggs. Exactly twenty L1 larvae were transferred to T175 flask containing 60 ml of sterile water and fed on alternative days with 60 μl of fish food (1 μg/μl). Larvae were inoculated with 1x10^7^/ml of either the WT *C*. *neteri*, the Δ*ompA* mutant or the Δ*ompA/ompA* complement. The WT and Δ*ompA* strains were transformed with the pRAM-mCherry plasmid [7] that conferred resistance to kanamycin (but did not possess a functional *ompA* gene). We also performed gnotobiotic infections with WT *C*. *neteri*, knockin mutants all expressing mCherry from a plasmid. In order to confirm that eggs were successfully sterilized, a T175 flask containing twenty L1 larvae were reared in identical fashion to mono-associations, albeit without bacterial supplementation. These larvae did not develop beyond the L2 stage, indicating our rearing process was free from contamination. To quantify bacteria, L4 larvae were collected, washed three times with 1X PBS, and then homogenized in 500 μl of 1X PBS and 50 μl of homogenate was plated on LB agar containing 50 μg/mL kanamycin. Similarly, adult mosquitoes were collected 3–4 days post emergence and bacterial infection was quantified in the same manner as larvae. In order to assess the growth of the mosquitoes, time to pupation and growth rate were observed. Time to pupation was determined by quantifying the number of pupae each day post hatching, while survival to adulthood was calculated by quantifying the number of L1 larvae that reached adulthood. The experiment was repeated three times.

### Reinfection of knockin mutants to mosquitoes

Knockin mutants were administered to 3–4 days adult *Ae*. *aegypti* in a sugar meal. These mosquitoes were reared under normal laboratory condition. Mosquitoes were fed with 1x10^7^ of WT or the Δ*ompA*::gentamicin strain for three days in 10% sucrose solution. After three days, mosquitoes were either administered sugar supplemented with gentamicin (50 μg/mL) or sugar without antibiotic. CFUs were determined at days 0, 2, 4, and 6 dpi by plating homogenized mosquitoes (N = 10) on LB agar. Similarly, the Δ*ompA*::mCherry and WT *C*. *neteri* were fed to mosquitoes and midguts were dissected to assess colonization of bacteria in the tissue. For visualization of bacteria, midguts were fixed in 1% paraformaldehyde (PFA) in 1X PBS for 30 minutes and permeabilized with 0.01% Triton X-100 in 1X PBS for 20 min. The tissues were stained with 1:250 diluted Phalloidin (Sigma) for 20 minutes and samples were washed twice with 1X PBS for 10 minutes. Finally, midguts were then stained with 1:500 diluted DAPI (Invitrogen) for 10 min. Samples were transferred to slides and mounted with ProLong^™^ Gold Antifade (Invitrogen). The slides were observed using a Revolve FL microscope (ECHOLAB).

## Supporting information

S1 Fig Midgut infection of *C*. *neteri* and *E*. *coli* in mono-associations of *Aedes* mosquitoes. Dissected gut tissue showing the conglomeration of bacterial cells when infected in mono-association in *Aedes* mosquitoes with *C*. *neteri* (A-C). However, *E*. *coli* (D-F) and Δ*ompA* (G-I) could not be seen in the midgut. Images were captured from the dissected midguts of different mosquitoes. Bacteria possessed the pRAM-mCherry plasmid which expressed the mCherry fluorescent protein.
(TIFF)

S2 Fig Phylogenetic analysis of WT and mutants. Multilocus sequence analysis according to [78] indicates isolates to be members of the *C*. *neteri* species; the MLST genes were amplified in the wild type isolate and two mutants as shown in the tree (red).
(TIFF)

S3 Fig Average CFUs of mosquito infection. Average CFU recovered in adult mosquitoes infected with *C*. *neteri* strains (WT, Δ*ompA* mutant and Δ*ompA/ompA* complement) reared in a mono-association using a gnotobiotic rearing approach. The uninfected mosquitoes were removed from the analysis. Box and whiskers show the 25^th^ and 75^th^ percentiles and the minimum and maximum values, respectively.
(TIFF)

S4 Fig Mono-association infection and biofilm assessment. L1 axenic larvae were infected with WT *C*. *neteri* (A), Δ*ompA*::mCherry (B) and Δ*ompA*::gentamicin (C) and adults gut were analysed for presence of bacterial conglomerations (biofilm formation). For each treatment, 15–18 midguts were screened. Scale bar 130 μm.
(PDF)

S1 Table gRNA sequence used in this study. (XLSX)

S2 Table Plasmids and bacterial strains used in this study. (XLSX)

S3 Table Primers used in this study. (XLSX)

S4 Table Infection and biofilm prevalence of mosquito midguts when reared in a mono-association. (XLSX)

S1 Appendix Multiple sequence alignment of WT and mutant *ompA* sequences of *C*. *neteri* and *E*. *coli*. (DOCX)
