# Supplementary figures and images for: CRISPR/Cas9-mediated gene deletion of the ompA gene in symbiotic Cedecea neteri impairs biofilm formation and reduces gut colonization of Aedes aegypti mosquitoes
Source: PLoS Negl Trop Dis. 2019 Dec 2;13(12):e0007883. doi: 10.1371/journal.pntd.0007883 (PMC6907859; doi:10.1371/journal.pntd.0007883)

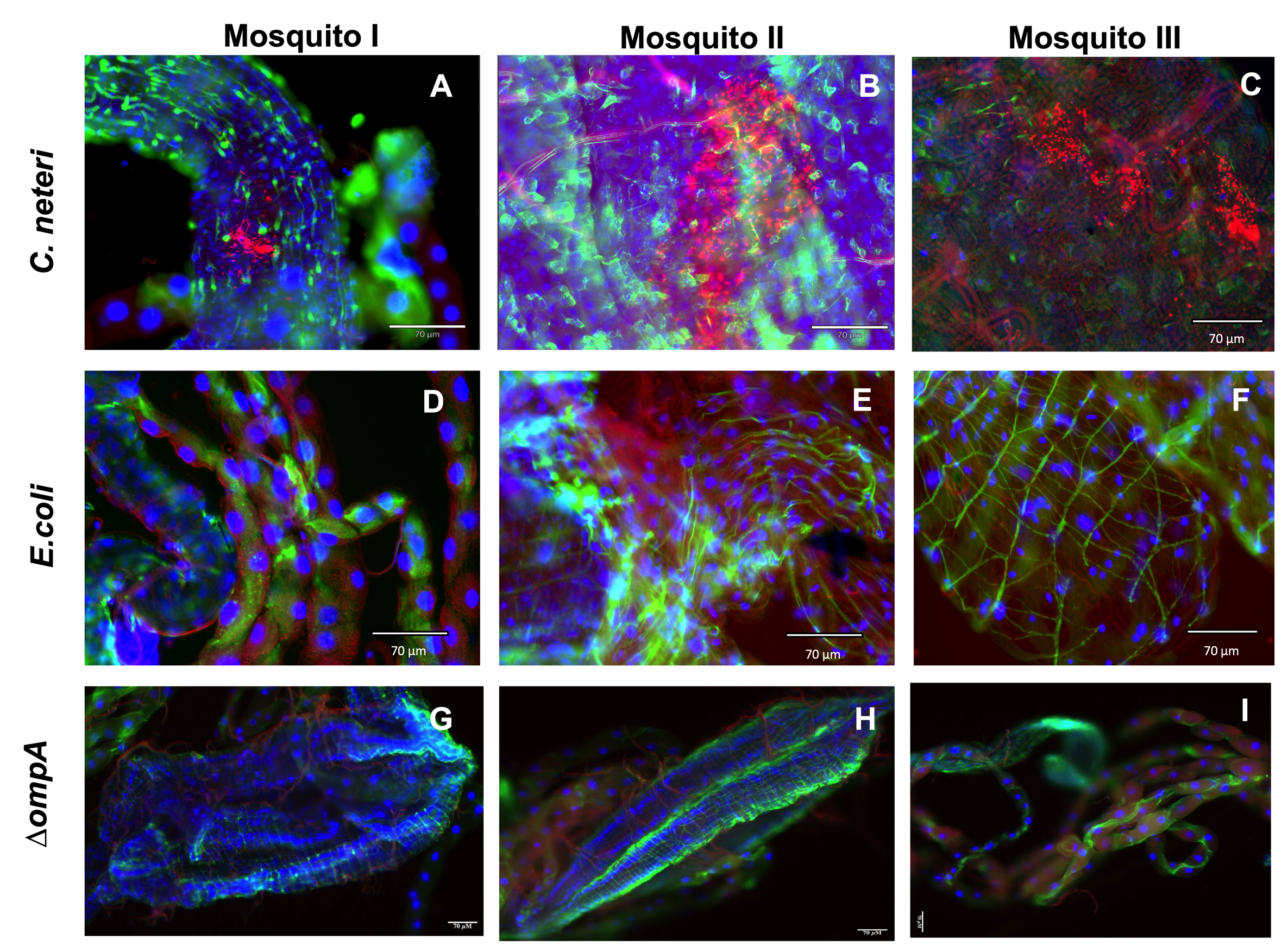

Supplement: S1 Fig — Dissected gut tissue showing the conglomeration of bacterial cells when infected in mono-association in Aedes mosquitoes with C. neteri (A-C). However, E. coli (D-F) and ΔompA (G-I) could not be seen in the midgut. Images were captured from the dissected midguts of different mosquitoes. Bacteria possessed the pRAM-mCherry plasmid which expressed the mCherry fluorescent protein. (TIFF) [file pntd.0007883.s001.tiff]

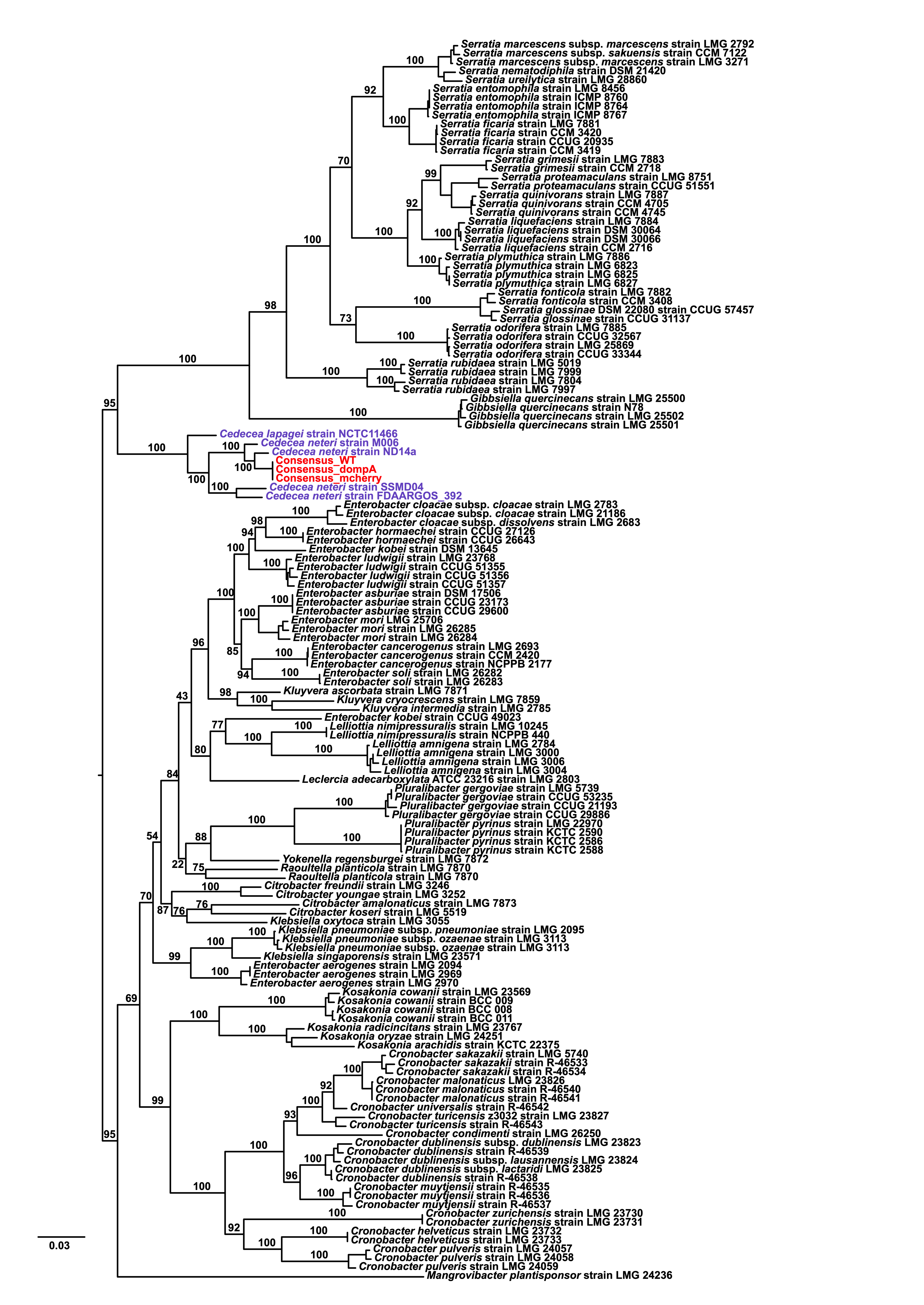

Supplement: S2 Fig — Multilocus sequence analysis according to [78] indicates isolates to be members of the C. neteri species; the MLST genes were amplified in the wild type isolate and two mutants as shown in the tree (red). (TIFF) [file pntd.0007883.s002.tiff]

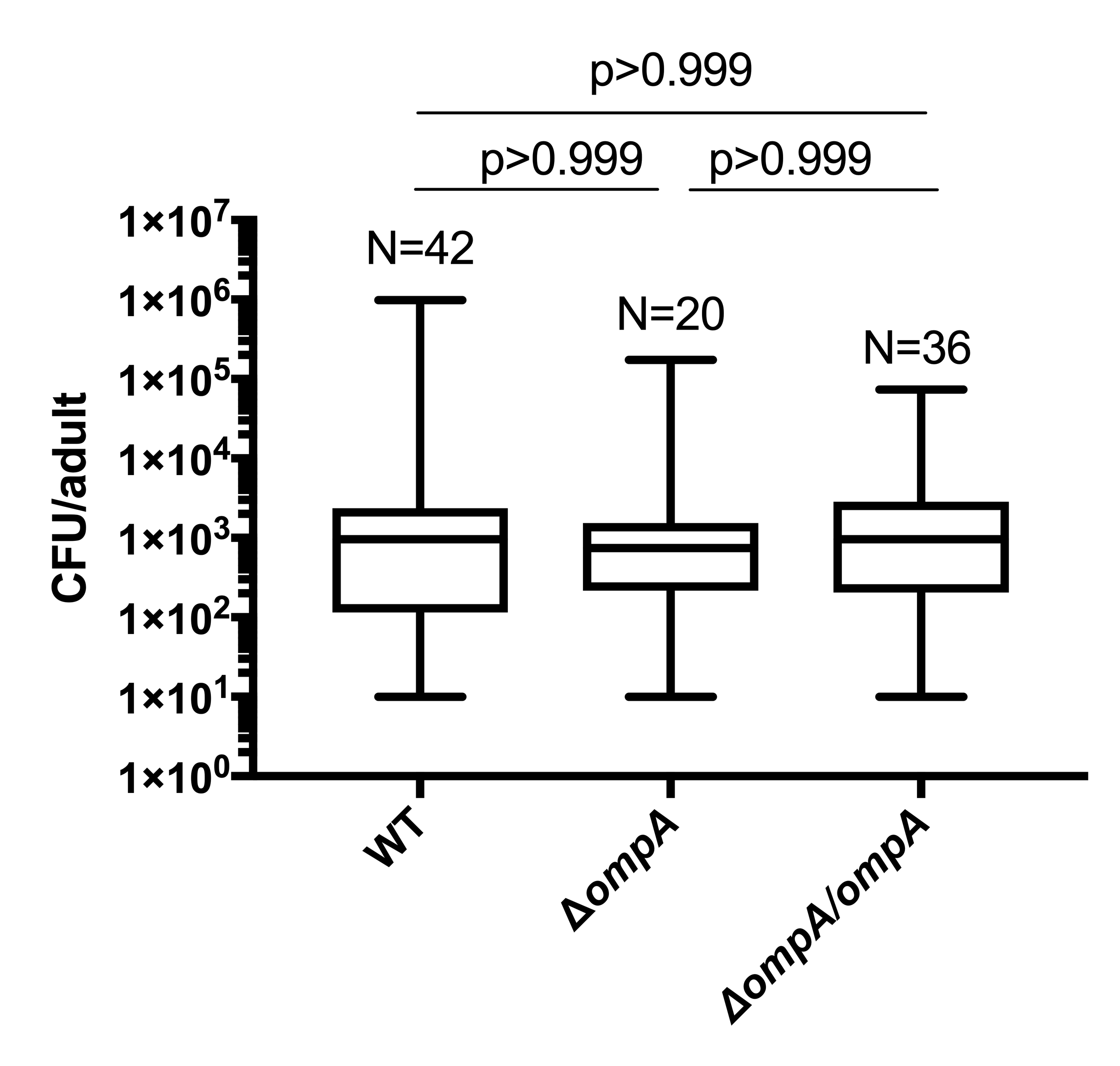

Supplement: S3 Fig — Average CFU recovered in adult mosquitoes infected with C. neteri strains (WT, ΔompA mutant and ΔompA/ompA complement) reared in a mono-association using a gnotobiotic rearing approach. The uninfected mosquitoes were removed from the analysis. Box and whiskers show the 25th and 75th percentiles and the minimum and maximum values, respectively. (TIFF) [file pntd.0007883.s003.tiff]

**A**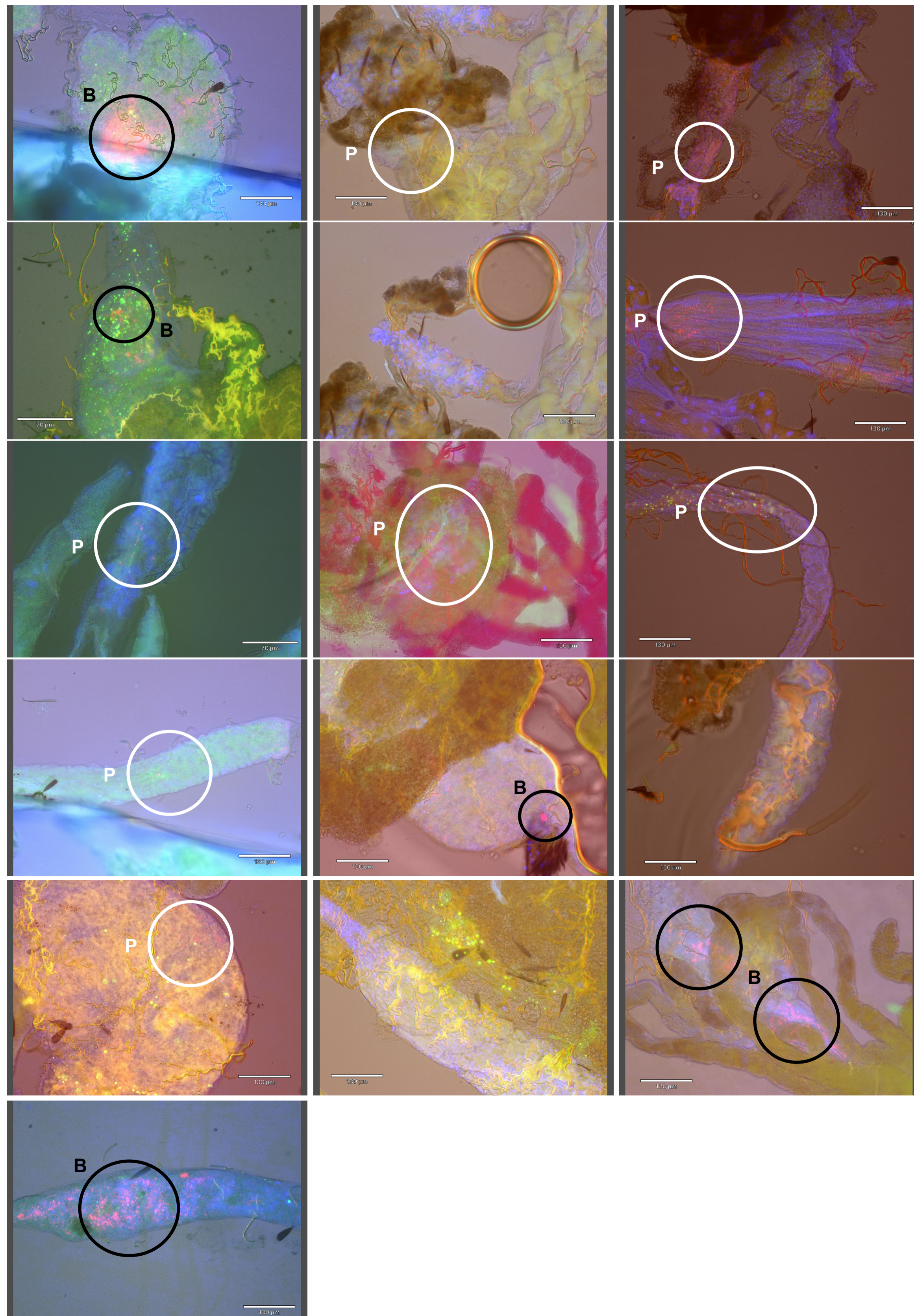**B**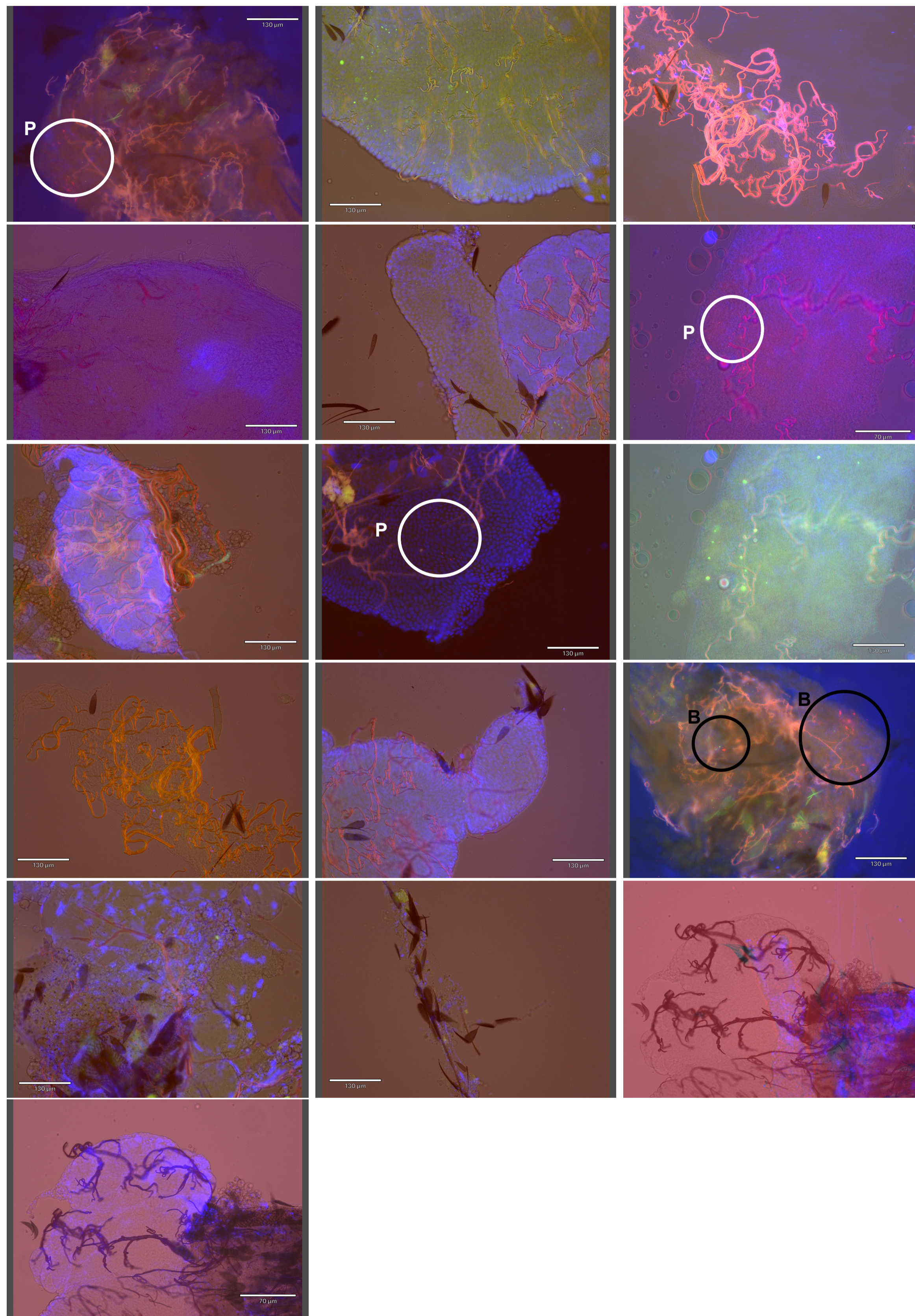**C**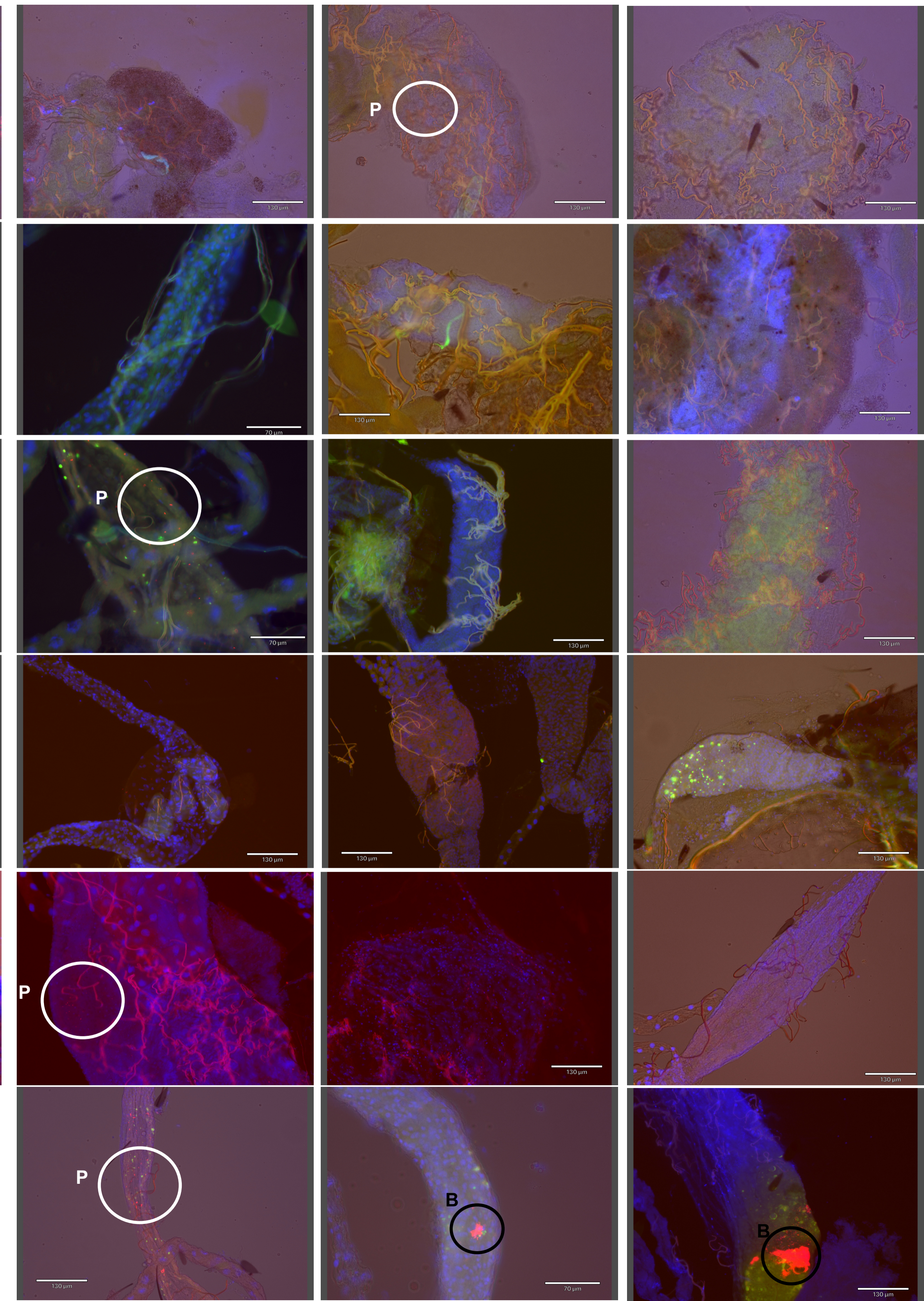

Supplement: S4 Fig — L1 axenic larvae were infected with WT C. neteri (A), ΔompA::mCherry (B) and ΔompA::gentamicin (C) and adults gut were analysed for presence of bacterial conglomerations (biofilm formation). For each treatment, 15–18 midguts were screened. Scale bar 130 μm. (PDF) [file pntd.0007883.s004.pdf]
