# Supplementary material for: CRISPR/Cas9-mediated gene deletion of the ompA gene in symbiotic Cedecea neteri impairs biofilm formation and reduces gut colonization of Aedes aegypti mosquitoes
Source: PLoS Negl Trop Dis. 2019 Dec 2;13(12):e0007883. doi: 10.1371/journal.pntd.0007883 (PMC6907859; doi:10.1371/journal.pntd.0007883)
Supplement: S1 Appendix — (DOCX) [file pntd.0007883.s009.docx]

**S1. Appendix**. Multiple sequence alignment of WT and mutant *ompA* sequences of *C. neteri* and *E. coli*.

Multiple sequence alignment *E. coli* WT *ompA* and mutant *ompA*

WT ATGAAAAAGACAGCTATCGCGATTGCAGTGGCACTGGCTGGTTTCGCTACCGTAGCGCAG

∆ompA ATGAAAAAGACAGCTATCGC----------------------------------------

********************

WT GCCGCTCCGAAAGATAACACCTGGTACACTGGTGCTAAACTGGGCTGGTCCCAGTACCAT

∆ompA ------------------------------------------------------------

WT GACACTGGTTTCATCAACAACAATGGCCCGACCCATGAAAACCAACTGGGCGCTGGTGCT

∆ompA ------------------------------------------------------------

WT TTTGGTGGTTACCAGGTTAACCCGTATGTTGGCTTTGAAATGGGTTACGACTGGTTAGGT

∆ompA ------------------------------------------------------------

WT CGTATGCCGTACAAAGGCAGCGTTGAAAACGGTGCATACAAAGCTCAGGGCGTTCAACTG

∆ompA ------------------------------------------------------------

WT ACCGCTAAACTGGGTTACCCAATCACTGACGACCTGGACATCTACACTCGTCTGGGTGGT

∆ompA ------------------------------------------------------------

WT ATGGTATGGCGTGCAGACACTAAATCCAACGTTTATGGTAAAAACCACGACACCGGCGTT

∆ompA ------------------------------------------------------------

WT TCTCCGGTCTTCGCTGGCGGTGTTGAGTACGCGATCACTCCTGAAATCGCTACCCGTCTG

∆ompA ------------------------------------------------------------

WT GAATACCAGTGGACCAACAACATCGGTGACGCACACACCATCGGCACTCGTCCGGACAAC

∆ompA ------------------------------------------------------------

WT GGCATGCTGAGCCTGGGTGTTTCCTACCGTTTCGGTCAGGGCGAAGCAGCTCCAGTAGTT

∆ompA ------------------------------------------------------------

WT GCTCCGGCTCCAGCTCCGGCACCGGAAGTACAGACCAAGCACTTCACTCTGAAGTCTGAC

∆ompA ------------------------------------------------------------

WT GTTCTGTTCAACTTCAACAAAGCAACCCTGAAACCGGAAGGTCAGGCTGCTCTGGATCAG

∆ompA ------------------------------------------------------------

WT CTGTACAGCCAGCTGAGCAACCTGGATCCGAAAGACGGTTCCGTAGTTGTTCTGGGTTAC

∆ompA ------------------------------------------------------------

WT ACCGACCGCATCGGTTCTGACGCTTACAACCAGGGTCTGTCCGAGCGCCGTGCTCAGTCT

∆ompA ------------------------------------------------------------

WT GTTGTTGATTACCTGATCTCCAAAGGTATCCCGGCAGACAAGATCTCCGCACGTGGTATG

∆ompA ------------------------------------------------------------

WT GGCGAATCCAACCCGGTTACTGGCAACACCTGTGACAACGTGAAACAGCGTGCTGCACTG

∆ompA ------------------------------------------------------------

WT ATCGACTGCCTGGCTCCGGATCGTCGCGTAGAGATCGAAGTTAAAGGTATCAAAGACGTT

∆ompA ------------------------------------------------------------

WT GTAACTCAGCCGCAGGCTTAA

∆ompA -TAACTCAGCCGCAGGCTTAA

********************

Alignment of *C. neteri* WT *ompA* and mutant *ompA* sequence

WT atgaaaaagacagctatcgcgattgcagtggcactggctggcttcgctaccgtagcgcag 60

∆ompA atgaaaaagacagctatcgcgattgcagtggcactggctggcttcgctaccgtagcgcag 60

************************************************************

WT gccgcaccgaaagataacacctggtatgcaggtggtaaactgggctggtctcagttccac 120

∆ompA gccgcaccgaaagataacacctggtatgcaggtggtaaactgggctggtctcagttccac 120

************************************************************

WT gataccggctggtataacaaggacctgcaaaacaacggtaacacccacgaaagccagctg 180

∆ompA gataccggctggtataacaaggacctgcaaaacaacggtaacacccacgaaagccagctg 180

************************************************************

WT ggcgctggtgccttcggtggttatcaggttaacccgtacgttggttttgaaatgggttac 240

∆ompA ggcgctggtgccttcggtggttatcaggttaacccgtacgttggttttgaaa-------- 232

****************************************************

WT gactggcttggccgtatgccttacaaaggcagcgtaactagcggtgccttcaaagctcag 300

∆ompA ------------------------------------------------------------ 232

WT ggcgtacagctgaccactaaactgggttacccaatcactgacgacctggacatctacacc 360

∆ompA ------------------------------------------------------------ 232

WT cgtctgggcggcatggtatggcgtgcagactctacaaacaccatcgctggtaaaaaccac 420

∆ompA ------------------------------------------------------------ 232

WT gacaccggtgtttccccagtattcgctggtggtgttgagtgggcagttacccgtgacatc 480

∆ompA ------------------------------------------------------------ 232

WT gctacccgtctggaataccagtgggttaacaacatcggtgatgcacagaccgttggcgtt 540

∆ompA ------------------------------------------------------------ 232

WT cgtcctgacaacggcatgctgagcgtaggtgtttcctaccgctttggccagcaggaagaa 600

∆ompA ------------------------------------------------------------ 232

WT gctgcaccagttgtagctccggctccagctccagctcctgaagtacagaccaaacacttc 660

∆ompA ------------------------------------------------------------ 232

WT actctgaagtctgacgtcctgttcaacttcaacaaagcaaccctgaaaccagaaggtcag 720

∆ompA ------------------------------------------------------------ 232

WT caggcactggatcagctgtacacccagctgagcaacctggatcctaaagatggttccgtt 780

∆ompA ------------------------------------------------------------ 232

WT gttgttctgggctacaccgaccgtatcggttccgagcagtacaacctgaaactgtctgaa 840

∆ompA --------------------------------------------------actgtctgaa 242

**********

WT aaacgtgcacagagcgttgttgattacctgatctctaaaggtatcccagcgaacaaaatc 900

∆ompA aaacgtgcacagagcgttgttgattacctgatctctaaaggtatcccagcgaacaaaatc 302

************************************************************

WT tctccacgtggcatgggcaaagctaacccagttaccggcaacacctgtgacaaagtagcg 960

∆ompA tctccacgtggcatgggcaaagctaacccagttaccggcaacacctgtgacaaagtagcg 362

************************************************************

WT cctaaagctaaactgatcgactgcctggctccagatcgtcgcgttgagatcgaagttaaa 1020

∆ompA cctaaagctaaactgatcgactgcctggctccagatcgtcgcgttgagatcgaagttaaa 422

************************************************************

WT ggtatcaaagaagttgtaactcagcctgcggcataa 1056

∆ompA ggtatcaaagaagttgtaactcagcctgcggcataa 458

**********************************
